# Deep Neural Network to Differentiate Brain Activity Between Patients With First-Episode Schizophrenia and Healthy Individuals: A Multi-Channel Near Infrared Spectroscopy Study

**DOI:** 10.3389/fpsyt.2021.655292

**Published:** 2021-04-15

**Authors:** Po-Han Chou, Yun-Han Yao, Rui-Xuan Zheng, Yi-Long Liou, Tsung-Te Liu, Hsien-Yuan Lane, Albert C. Yang, Shao-Cheng Wang

**Affiliations:** ^1^Department of Psychiatry, China Medical University Hsinchu Hospital, Hsinchu, Taiwan; ^2^Department of Photonics, National Chiao Tung University, Hsinchu, Taiwan; ^3^Department of Biological Science and Technology, National Chiao Tung University, Hsinchu, Taiwan; ^4^MediaTek Inc., Hsinchu, Taiwan; ^5^Graduate Institute of Electronics Engineering, National Taiwan University, Taipei, Taiwan; ^6^Graduate Institute of Biomedical Sciences, China Medical University, Taichung, Taiwan; ^7^Department of Psychiatry and Brain Disease Research Center, China Medical University Hospital, Taichung, Taiwan; ^8^Department of Psychology, College of Medical and Health Sciences, Asia University, Taichung, Taiwan; ^9^Institute of Brain Science, National Yang-Ming University, Taipei, Taiwan; ^10^Digital Medicine Center, National Yang-Ming University, Taipei, Taiwan; ^11^Brain Medicine Center, Taoyuan Psychiatric Center, Taoyuan, Taiwan; ^12^Department of Forensic and Addiction Psychiatry, Jianan Psychiatric Center, Ministry of Health and Welfare, Tainan, Taiwan; ^13^Department of Mental Health, Johns Hopkins Bloomberg School of Public Health, Baltimore, MD, United States; ^14^Department of Medical Laboratory Science and Biotechnology, Chung Hwa University of Medical Technology, Tainan, Taiwan

**Keywords:** deep neural network, near infrared spectroscopy, schizophrenia, machine learning, fNIRS, deep learning

## Abstract

**Backgrounds:** Reduced brain cortical activity over the frontotemporal regions measured by near infrared spectroscopy (NIRS) has been reported in patients with first-episode schizophrenia (FES). This study aimed to differentiate between patients with FES and healthy controls (HCs) on basis of the frontotemporal activity measured by NIRS with a support vector machine (SVM) and deep neural network (DNN) classifier. In addition, we compared the accuracy of performance of SVM and DNN.

**Methods:** In total, 33 FES patients and 34 HCs were recruited. Their brain cortical activities were measured using NIRS while performing letter and category versions of verbal fluency tests (VFTs). The integral and centroid values of brain cortical activity in the bilateral frontotemporal regions during the VFTs were selected as features in SVM and DNN classifier.

**Results:** Compared to HCs, FES patients displayed reduced brain cortical activity over the bilateral frontotemporal regions during both types of VFTs. Regarding the classifier performance, SVM reached an accuracy of 68.6%, sensitivity of 70.1%, and specificity of 64.6%, while DNN reached an accuracy of 79.7%, sensitivity of 88.8%, and specificity of 74.9% in the classification of FES patients and HCs.

**Conclusions:** Compared to findings of previous structural neuroimaging studies, we found that using DNN to measure the NIRS signals during the VFTs to differentiate between FES patients and HCs could achieve a higher accuracy, indicating that NIRS can be used as a potential marker to classify FES patients from HCs. Future additional independent datasets are needed to confirm the validity of our model.

## Introduction

Schizophrenia (SZ) is a chronic psychiatric disorder characterized by psychotic symptoms, negative symptoms, and cognitive deficits and poses considerable burdens to society (1). Therefore, accurate diagnosis and early intervention are critical (2, 3). In clinical practice, schizophrenia is diagnosed by clinicians using diagnostic criteria from the Diagnostic and Statistical Manual of Mental Disorders (DSM-5), based on patient reports of symptoms, observation of behavior and functional changes; however, traditional clinical practice might be confounded because patients with SZ may deny their symptoms, and even experienced psychiatrists may have difficulty differentiating SZ from other mental illnesses (i.e., psychotic bipolar disorder) owing to similar symptomologies at acute stage (4).

To overcome these limitations of clinical interviews-based diagnosis of psychiatric disorders, many studies have attempted to develop objective biomarkers that can improve the accuracy of diagnosis and the ability to predict a patient's response to treatment and prognosis. Among a variety of neuroimaging modalities, functional near infrared spectroscopy (fNIRS) is a functional neuroimaging tool that measures the spatio-temporal neural activity of the brain non-invasively. Compared to existing imaging techniques, such as positron emission tomography (PET), single-photon emission computed tomography (SPECT), and magnetic resonance image (MRI), fNIRS is easier to administer, low-cost, and provides fair temporal and spatial resolutions (5). Many previous fNIRS studies reported reduced brain activity over the bilateral frontotemporal regions during various cognitive tasks in patients with SZ compared to controls [reviewed by Koike et al. (6) and Chou et al. (7)].

Recently, many studies have attempted to accurately classify patients with heterogeneous mental disorders. For instance, several studies used machine learning (ML) methods to accurately differentiate patients with SZ and healthy individuals with structural or functional neuroimaging tools and showed promising results (8). ML methods are capable of representing latent features of structural or functional changes in the brain, and this allows for better representation of SZ-related processes. Among ML methods, support vector machines (SVMs) are mostly adopted. SVM is an ML method which estimates a hyperplane with an optimal margin that could provide the best separation between two classes, which is determined by the maximum distance from any data point. Once defined, this hyperplane is used to classify the data (8, 9).

Recently, deep learning (DL) methodology such as deep neural network (DNN) has significantly improved the representation learning and classification in various areas such as speech recognition, natural image classification, and text mining (9). Two main features have made DNN unique compared to SVM. First, DNN is capable of data-driven automatic feature learning, which enables to remove the subjectivity in selecting the relevant features when there are too many features or no prior knowledge in selecting features. Second, by applying a hierarchy of non-linear layers, DNN can analyze complicated data patterns (8). Recently, DL methods have been applied in medical image analyses with promising results, including characterizing patterns of brain imaging data in patients with neurocognitive disorders (10–13) and schizophrenia (9, 14). However, most previous studies analyzed MRI data.

In the present work, we aimed to discriminate between patients with first-episode schizophrenia (FES) and healthy controls (HCs) on the basis of brain cortical activity during a verbal fluency test (VFT) measured using NIRS. We focused on the bilateral frontotemporal regions. We compared classification accuracies for two different machine learning methods: SVM and DNN. To the best of our knowledge, this is the first study using deep learning to automatically differentiate FES from HC based on brain cortical activity features.

## Materials and Methods

### Study Subjects

A total of 33 patients with FES (18 men and 15 women; mean age [SD] =29.1 [6.4] years) were recruited at the Department of Psychiatry in Taichung Veterans General Hospital. Patients who fulfilled the criteria for SZ listed in the DSM-5 were recruited and the diagnoses were validated using the Mini International Neuropsychiatric Interview (MINI) (15) by board-certified psychiatrists (P.H.C). All patients were experiencing their first episode of psychosis and had received no more than 12 weeks of previous antipsychotic medication (16, 17). Thirty-four HCs (17 men and 17 women; mean age [SD] = 28.2 [9.9] years) were recruited and screened using the MINI. All study participants were right-handed, which was assessed by the Edinburgh Inventory (18). Other characteristics such as education level, VFT performance of study subjects, as well as the age of onset and duration of illness of FES patients were also recorded. Subjects were excluded if they had a history of substance abuse or dependence, intellectual disability, neurological disorders, or a medical condition that may affect brain function. This study complied with the Declaration of Helsinki, and all participants received a complete explanation of the study and provided written informed consent. This study was approved by the Institutional Review Board of Taichung Veterans General Hospital (approval No. CF13044).

### Clinical Assessments

We used the Positive and Negative Syndrome Scale (PANSS) (19) to evaluate psychiatric symptoms of the FES patients on the same day as the NIRS measurements. Patient antipsychotic doses are presented as chlorpromazine-equivalent doses (20, 21).

### Verbal Fluency Test

Patients received 160-s block-design VFTs (both letter and category version) which has been adopted in many previous fNIRS studies (17, 22–26). There were three different time periods for the VFT: a 30-s pre-task period, a 60-s task period, and a 70-s post-task period. In the pre- and post-task periods, patients were asked to repeatedly count from one to five to control for and remove task-related motion artifacts. For the 60-s task period, study participants were instructed to say words that started with a phonological syllable presented by NIRS machine. In the letter fluency test (LFT), there three continuous 20-s sub-periods, which were initiated by a single Chinese syllable selected from nine options (first, /ㄅ(b)/, /ㄆ(p)/, or /ㄉ(d)/; second, /ㄊ(t)/, /ㄌ(l)/, or /ㄋ(n)/; third, /ㄇ(m)/, /ㄈ(f)/,or /ㄘ(dz)/). We chose these syllables based on their frequencies at the beginning of Chinese words. For the category fluency test (CFT), subjects were asked to produce as many words based on a given semantic cue for 20 s each (first: “fish,” “birds,” or “insects”; second: “sweets,” “vegetables,” or “fruits”; third: “vehicles,” “home appliances,” or “stationery items,”). Before beginning each task session, subjects were instructed on how to generate correct answers for VFTs. Each subject practiced three times to ensure that they understood the tests.

### NIRS Instrument

**Figure 1:**
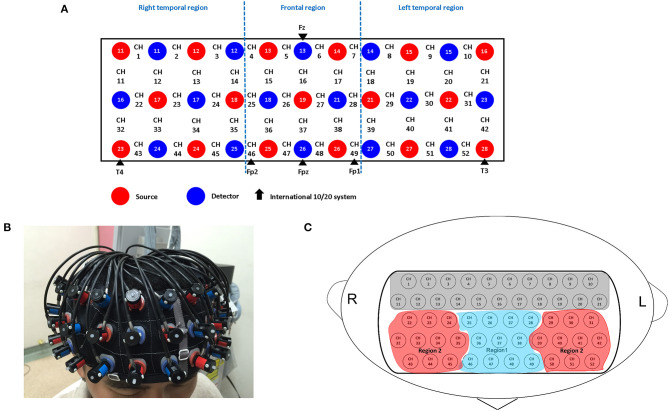
Probe setting and measurement points for 52-channel NIRS. **(A)** The localizations of channels are set based on the international 10–20 electroencephalography system. Red and blue circles, respectively indicate the emitter and detector of the near-infrared light. **(B)** Probes with thermoplastic 3 × 11 shells are placed over the bilateral frontotemporal area. **(C)** Two regions of interest (Regions 1 and 2) in the present study. Region 1 consists of 11 channels (ch 25–28, ch 36–38 and ch 46–49); Region 2 consists of 20 channels; Right: (ch 22–24, ch 32–35 and ch 43–45); Left: (ch 29–31, ch 39–42 and ch 50–52).

A 52-channel NIRS instrument (ETG-4000; Hitachi Medical Co., Tokyo, Japan) was used to measure changes in concentrations of oxygenated hemoglobin [oxy-Hb] of the brain in the present study. The NIRS probe attachments are thermoplastic 3 × 11 shells set, comprising 52 channels (Figure 1). The lowest probe line was set along the Fp1–Fp2 line, as defined by the international 10–20 system used in electroencephalography. The NIRS instrument measures changes in both [oxy-Hb] and [deoxy-Hb] (in mM) using two wavelengths (695 and 830 nm) of near-infrared light. The calculations were based on the Beer–Lambert law (27). We recorded the changes of [oxy-Hb] from baseline to the activation period and relative changes in [oxy-Hb] assessed with units of mM·mm. The data sampling rate for NIRS instrument was 0.1 s. A moving average methodology using a 5-s window width was applied and any motion artifacts were automatically detected and rejected by the machine (28).

The spatial information for each channel was estimated by using data from the Functional Brain Science Laboratory at Chuo University in Japan (29) based on the LONI Probabilistic Brain Atlas (LPBA40) (30). Because previous study indicated that [oxy-Hb] had stronger correlations with fMRI blood-oxygenation level-dependent signals (31), we used it as an indicator of brain cortical activity.

### NIRS Signals and Feature Selection

Similar to Takizawa et al.'s study (32), two regions of interest (ROIs) were selected (Figure 1): the frontal region (R1, 11 channels) and the bilateral temporal region (R2, 20 channels). The changes in [oxy-Hb] and [deoxy-Hb] in the channels of these two respective regions of interest were averaged and transformed into representative “Region 1 (R1)” and “Region 2 (R2)” NIRS signals for each individual. According to the LBPA40 (30), the “Region 1 (R1)” NIRS signal consisted of signals from channels located approximately in the fronto-polar and dorsolateral prefrontal cortical regions, while the “Region 2 (R2)” NIRS signal consisted of signals from channels located approximately in the bilateral ventro-lateral prefrontal cortex and the superior and middle temporal cortical regions.

**Figure 2:**
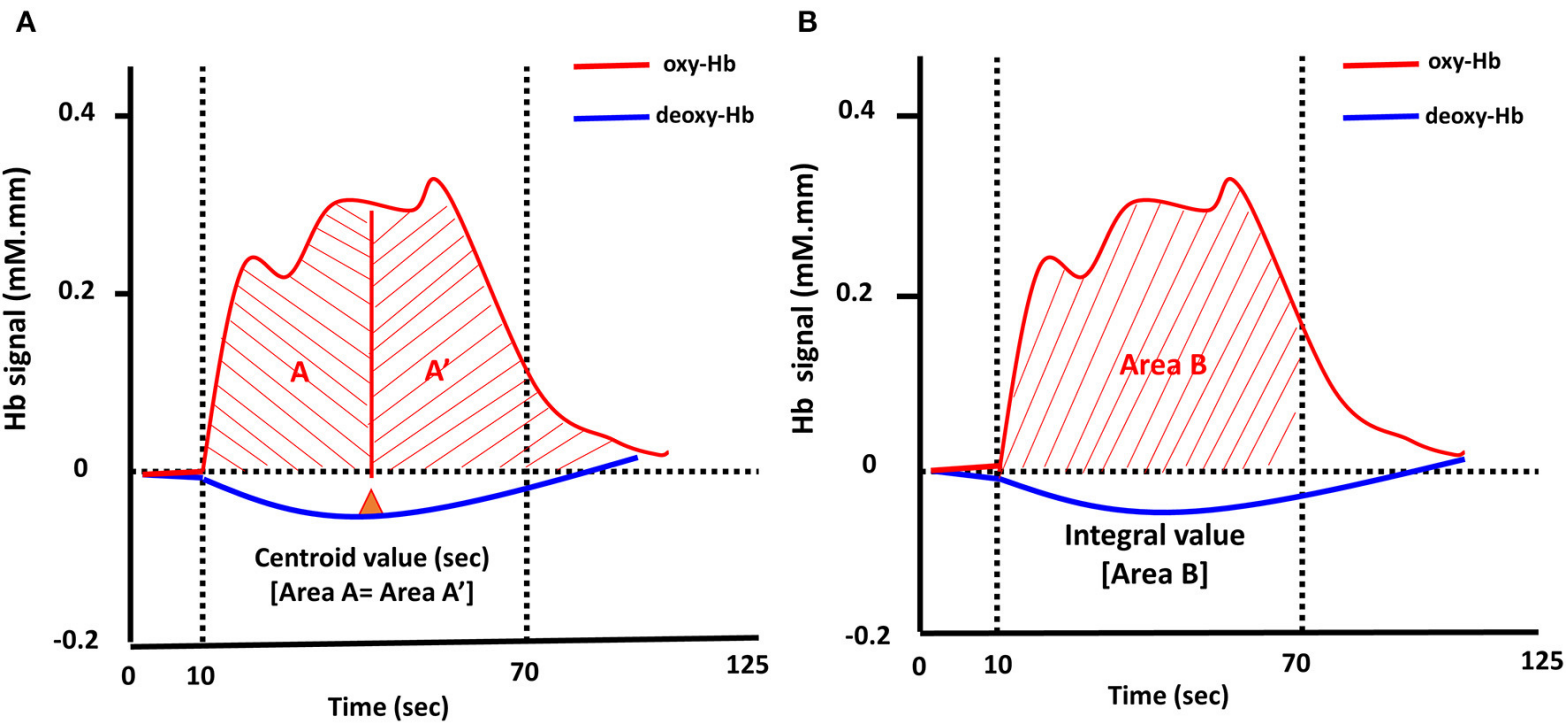
Typical time-course pattern of NIRS signals during the VFT. The “centroid value” is defined by a perpendicular line from the centroid of the brain activation area during the task period **(A)**. The “integral value (Area B)” indicated the brain activity during the test **(B)**; Oxy-Hb, oxygenated hemoglobin; deoxy-Hb, deoxygenated hemoglobin.

Two visual indices, integral and centroid value, of the bilateral frontotemporal regions during LFT and CFT (Figure 2) were generated automatically from the NIRS machine by evaluating the hemodynamic changes in [oxy-Hb] of the 10-s pre-task, 60-s task, and 55-s post-task period from the original 160-s VFTs. Details regarding the definition of integral and centroid value can be found elsewhere (32). In brief, integral value was calculated using the hemodynamic response of [oxy-Hb] during the 60-s activation task period by averaging the signal from channels within each region; the centroid value is an index of time-course changes throughout the VFT, with periods representing the timing of the hemodynamic response. The centroid value is indicated by the time shown with a perpendicular line from the centroid of the [oxy-Hb] signal change area during the entire task periods [from 0 (s) to 125 (s) [= 10 (s) + 60 (s) + 55 (s)]]; the integral value describes the size of the hemodynamic response during the 60-s test period (32). Therefore, a total of eight datasets were collected (integral and centroid values of R1 and R2, during an LFT and CFT, respectively).

### Deep Neural Network

**Figure 3:**
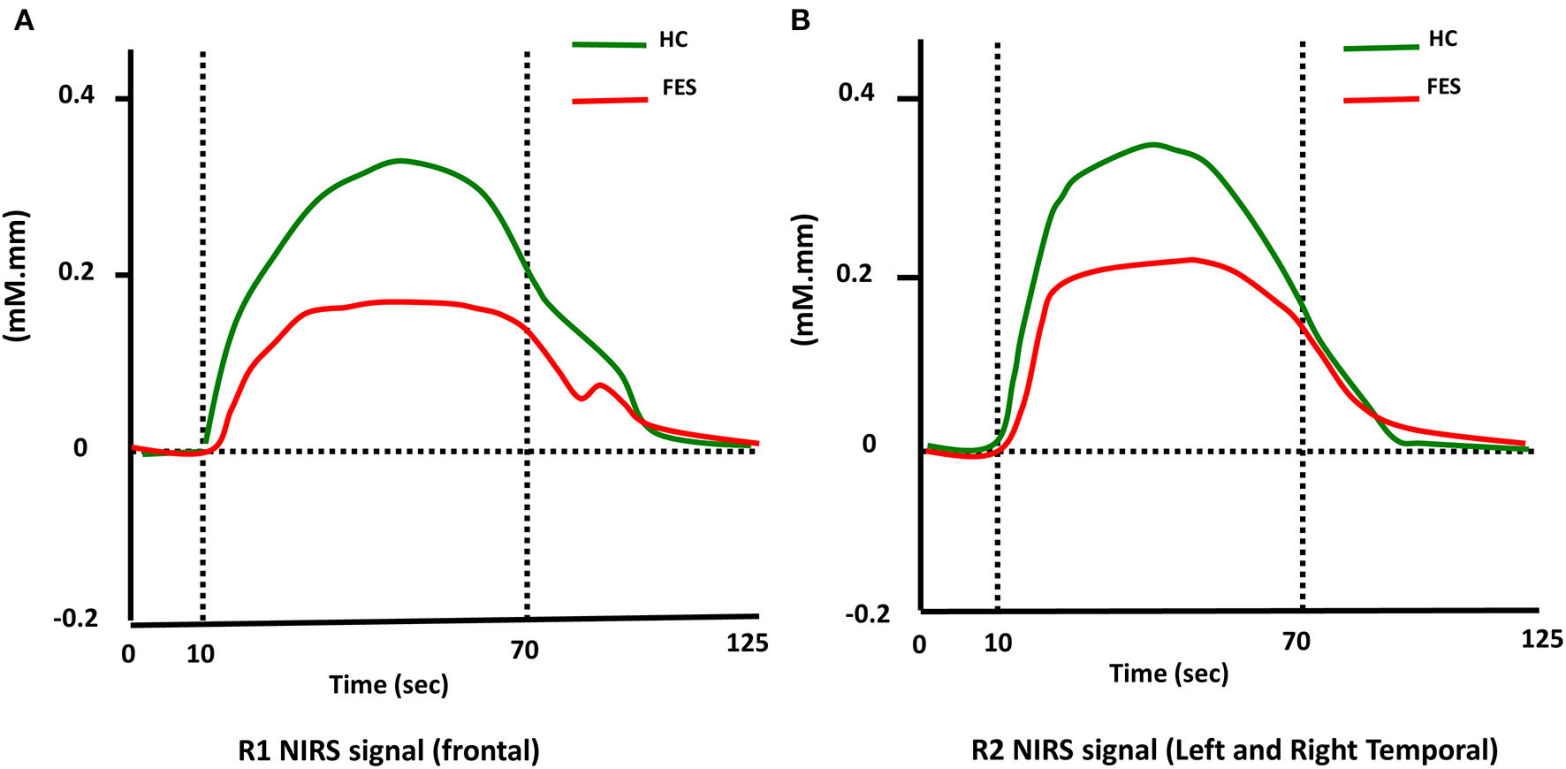
Time courses of the hemodynamic responses of [oxy-Hb] in Region 1 (R1) and Region 2 (R2) in FES and HC groups. **(A)** and **(B)** show the time courses of the hemodynamic responses in R1 (frontal region) and R2 (temporal region), respectively.

A DNN was utilized as a classifier to discriminate the patients with schizophrenia from healthy control (HC). The network had eight features as inputs (which included the integral and centroid values of the frontal and temporal regions during the two types of VFTs. in the NIRS signal) (Figure 3). The topology of this classifier is a fully connected, four-layer, feedforward network, which comprises two hidden layers with 512 neurons for each layer. The activation function of all neurons in the network is the rectified linear unit (ReLU) function. The network outputs 2 indices in the last layer that the index with larger value indicates positive (FES) or negative (HC).

#### Training of DNN

This DNN was trained with supervised learning since labeled data (FES or HC) were given. During training, stochastic gradient descent (SGD) was employed for optimizing the parameters; error gradients were propagated backwards through layers, which was backpropagation. Each parameter in the network was randomly initialized and adjusted according to its corresponding gradient to loss to minimize the error between the predicted results and the labeled data. In addition, the dropout technique was incorporated to avoid overfitting, and the dropout rate was 0.18. Here a criterion was set that the learning stage was stopped when the value of cost function changes little through epochs, and according to experiences, the learning duration was expected to be 100 to 300 epochs.

In the training procedure, cross-validation was required since the performance of the DNN was evaluated here by validation accuracy, practically, which was shuffling the 67-example dataset (33 FES patients and 34 HCs) at first and then dividing it into seven groups. In each turn of cross-validation, one of the groups was used as the validation set and the other six groups were training sets, and the validation set contained 10 samples, and the training set the other 57 samples.

### Support Vector Machines

In the present study, Support Vector Machine (SVM) was used to compare the performances with which by deep learning. In machine learning, SVM is a supervised-learning method that learns model from labeled training data, and has been used for classification of patients with different psychiatric diseases (33). The SVM methodology has been detailed elsewhere (34). Given a training dataset for classification, the SVM algorithm optimizes for the support vectors that is a subset of training data and represents a hyperplane dividing the training data into their labeled categories with gaps as wide as possible. The prediction is then made by evaluating the decision function with test data as input and support vectors as parameters. A model of SVM with hyper parameter c = 1.0 and with radial basis function (RBF) kernel (γ = 1/Nt, Nt: the number of training examples) is built to run the SVM algorithm. The formation of the input data and processing of cross-validation are exactly the same to ensure consistency and fair comparisons.

### Statistical Analysis

Firstly, Kolmogorov–Smirnov or Shapiro-Wilk tests were used to examine the distribution of the data. Basic characteristics in each group were compared using Student's *t*-tests for continuous variables and *X*^2^ test for categorical variables. When the data was not normally distributed, a non-parametrical analysis, that is, Spearman's rho was used to examine correlations and the Mann–Whitney *U*-test was used to compare means. Otherwise, the *t*-test was used to compare means and Pearson's correlation coefficient was used to examine correlations. *P*-value < 0.05 was defined statistically significant. All statistical analyses were performed using STATA version 15.1. DNN and SVM were performed using Python with open source library packages including Keras, scikit-learn, and TensorFlow.

## Results

### Demographic Characteristics

The study participants' demographic characteristics are presented in Table 1. There were no significant differences between the HC and FES groups in terms of age, sex, or education. However, the HC group had significantly better performance on the LFT (HC group, mean= 14.0, SD = 0.8; FES group, mean = 9.7, SD=0.8, *P* < 0.001) and CFT (HC group, mean = 17.5, SD = 0.8; FES group, mean = 12.1, SD = 0.7, *P* < 0.001) compared to the FES group.

**Table 1:** Characteristics of study participants.

	**FES (*N* = 33)**	**HC (*N* = 34)**	**Statistics/analyses**	***P*-value**
Age	29.1 (6.4)	28.2 (9.9)	*T* = 0.42	0.68
Education (graduate/undergraduate/high school degrees)	7/18/8	3/27/4	X^2^ test	0.09
Right handed	33	33	X^2^ test	1
Gender(M/F)	(18/15)	(17/17)	X^2^ test	0.71
LFT performance	9.7 (0.8)	14.0 (0.8)	*t* = −3.80	*P* < 0.001
CFT performance	12.1 (0.7)	17.5 (0.8)	*t* = −5.03	*P* < 0.001
Onset age	27.2 (6.1)			
DOI (week)	102.8 (126.5)			
**PANSS**				
Positive	16.8 (5.3)			
Negative	17.5 (5.5)			
General psychopathology	33.9 (7.6)			
Total	68.1 (14.7)			
antipsychotics	426.9 (236.1)			

*FES, first-episode schizophrenia; HC, healthy control; LFT, letter version of verbal fluency test; CFT, category version of verbal fluency test; DOI, duration of illness; PANSS, positive and negative symptom scale*.

### Comparison of Hemodynamic Response of ROIs Across Clinical Groups

As shown in Table 2, during the LFT, significantly smaller integral values of [oxy-Hb] in the SZ than the HC group (R1: *P* < 0.001, *t* = 3.859; R2: *P* = 0.003, *t* = 3.047) were noted. On the other hand, there were no significant differences between two groups with regard to centroid values in both regions (R1: *P* = 0.667, *t* = −0.433; R2: *P* = 0.138, *t* = −1.515). During the CFT, smaller integral values of [oxy-Hb] in the SZ than the HC group were noted in both regions (R1: *P* = 0.015, *t* = 2.507; R2: *P* = 0.006, *t* = 2.845), and no significantly different centroid values between the two groups (R1: *P* = 0.528, *t* = −0.635; R2: *P* = 0.796, *t* = −0.259).

**Table 2:** Comparison of frontal or temporal integral and centroid value of NIRS signals between FES and HC groups^a^.

	**Frontal region (R1)**				**Temporal region (R2)**			
	**Integral**	***P*-value**	**Centroid**	***P*-value**	**Integral**	***P*-value**	**Centroid**	***P*-value**
LFT								
HC group	131.9 (12.0)	**0.0003**	56.5 (1.4)	0.6633	204.7 (17.4)	**0.0033**	56.8 (0.7)	0.1299
FES group	47.6 (18.4)		57.8 (2.8)		114.9 (23.9)		60.7 (2.4)	
CFT								
HC group	82.5 (12.8)	**0.0147**	57.2 (1.8)	0.5279	178.7 (17.8)	**0.0059**	60.7 (1.0)	0.7950
FES group	36.3 (13.3)		59.3 (2.8)		100.2 (21.2)		61.2 (1.6)	

a *The unit for NIRS signal is (mM.mm). Statistical significance was marked with bold character*.

*FES, first-episode schizophrenia; HC, healthy control; LFT, Letter version of Verbal Fluency Test; CFT, Category version of Verbal fluency Test*.

### Classification Performance of DNN and SVM

In DNN, the topology of the network is determined by the experiments on network with different number of hidden layers and different number of neurons per layer, as shown in Table 3. A larger or deeper network generally performs better but harder to train. According to the results from the experiments, a 4-hidden-layer and 512-neurons-per-layer neural network was selected in the present study.

**Table 3:** Nnetwork topology demonstrating comparison accuracy (%) of DNN.

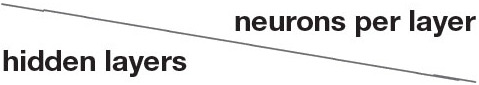	**64**	**128**	**256**	**512**	**1,024**
1	55.7	58.2	63.6	62.0	69.4
2	61.4	65.1	74.2	79.1	78.8
3	60.2	65.1	76.0	78.0	79.1
4	61.7	66.5	77.1	**79.7**	79.4
5	51.7	63.4	72.9	79.1	78.6

*DNN, deep neural network. Bold value indicated the best accuracy of performance*.

To reduce the effect of randomness, the cross-validation accuracy in each training set group is the average of the accuracies obtained by training and testing the network with different initializations five times. Therefore, the classification accuracy of DNN was 79.7%, sensitivity of 88.8%, and specificity of 74.9%. On the other hand, the result of classification accuracy using the eight features analyzed by SVM was 68.6%, sensitivity of 70.1.8%, and specificity of 64.6%.

### Correlational Analyses

During the LFT, there was a significant negative correlation between R1 integral values and PANSS general psychopathology score (rho = −0.371, *P* = 0.034). In addition, there were significant negative associations between R2 integral values and PANSS negative (rho = −0.551, *P* = 0.001) and general psychopathology scores (rho = −0.433, *P* = 0.012). With regard to CFT, there was a significant positive correlation between R1 integral values and antipsychotic dosage (rho = 0.403, *P* = 0.020). In addition, there was significantly negative associations between R2 integral values and PANSS general psychopathology scores (rho = −0.501, *P* = 0.003).

## Discussion

To our knowledge, this study is the first to evaluate the classification performance of artificial intelligence to distinguish patients with FES and HCs using NIRS signals. In the present study, we employed SVM and DNN methods to automatically differentiate FES patients from HCs. The main findings can be summarized as follows. (1) We reached a fair discrimination accuracy using SVM on integral and centroid values of R1 and R2 during both types of VFTs (68.6%). (2) DNN achieved modestly higher predictive performance than the SVM approach (79.7%). (3) Compared to HCs, there was decreased cortical activity in FES patients during the LFT but not the CFT, indicating that deficits in cortical activity during phonemic processing may occur early in the course of SZ.

### Comparison of Classification Performance Between DNN and SVM

In the present study, we found classification accuracy of DNN is better than SVM, which is consistent with many previous MRI studies demonstrating superiority of DNN over SVM (9, 14, 35). SVM, a shallow-structured architecture, are effective in solving many simple or well-constrained problems. However, several recent studies have demonstrated the benefits of using deep structures. DNN may be more robust in the wide variety of functions that can be parameterized by composing weakly non-linear transformations. DNN allows a system input to be compositing from raw data, thus allowing automatic discovery of the representations required for machine learning tasks (36). Finally, the appeal of hierarchical representations and the potential for combining unsupervised and supervised methods also contribute to the use of deep neural networks (9). However, in this study, we did not explore all possible deep learning advantages, such as the use of input data without feature extraction. Instead, we selected the features generated by NIRS machine. Nevertheless, our results showed that when using NIRS signals, the DNN-based model can achieve better classification performance than SVM model.

### Comparison With Previous fNIRS/MRI Studies Using Deep Learning or Machine Learning

Until now, there have been few NIRS studies using ML or DL method to classify patients with SZ and healthy individuals (37, 38). Li et al. (37) recruited a large sample of 120 SZ patients and 120 HCs and measured the hemoglobin response in the prefrontal cortex during the VFT using a multichannel NIRS instrument. They used PCA-based feature selection for data extracted from three types of NIRS data in each channel, and they achieved a maximum accuracy of 85.83% and an overall mean accuracy of 83.37% using SVM classifier. Yang et al. (38) measured the functional connectivity strength (FCS) as features derived from an individual channel during the VFT in 100 patients with schizophrenia and 100 healthy controls, and applied principal component analysis. They found that FCS from three channels on the medial prefrontal and left ventrolateral prefrontal cortices rendered accuracy as high as 84.67%, sensitivity at 92.00%, and specificity at 70%. However, due to the differences in study population recruited, usage of fNIRS features, and machine learning algorithms, it was difficult for us to directly compare these two studies.

On the other hand, there have been many structural or functional MRI studies using machine learning (e.g., SVM) technique reporting heterogeneous classification performances (with accuracies ranging from 60 to over 95%) in the classification of patients with chronic or first episode SZ against healthy individuals [reviewed by (8)]. However, there have been few MRI studies using deep learning to discriminate patients with schizophrenia and healthy controls. In the structural MRI study conducted by Pinaya et al. (9), the authors compared classification performance of deep belief network (DBN) and SVM between patients with schizophrenia and healthy individuals. They found DBN was slightly more accurate as a classifier (accuracy = 73.6%) than the SVM (accuracy = 68.1%) between patients with SZ and healthy individuals. However, the error rate of the DBN in classifying patients with first-episode psychosis (FEP) was 56.3%. In another study conducted by Vieira et al. (39), they used DNN to analyze a total of 956 participants (514 FEP and 444 HCs) and found that the best accuracies (70%) were achieved when DNN was applied compared to that when SVM was used (61.3%). In the present study, we demonstrated the classification accuracy of DNN (79.7%) was superior to that of SVM (68.6%), a finding similar to that reported by Vieira et al. However, Vieira et al. found it was difficult for the DNN models generalized to other sites, indicating that detection of individuals in the early stages of psychosis is more challenging. In the present study, we did not test our DNN model in another independent dataset, and future study using fNIRS dataset from other sites to test our DNN model is warranted.

### Comparison of the Results of Correlational Analyses With Previous NIRS Studies

Similar to previous NIRS studies, we found that cortical activities over bilateral frontotemporal regions were negatively correlated with PANSS negative (17, 40) or general psychopathology scores (17, 28) during the both versions of VFTs. However, it is interesting to note that there was a significant positive correlation between frontal activity (R1 integral value) and antipsychotic dosage during the CFT, which has never been reported before. Antipsychotic treatment has been shown to improved cognitive function in first-episode and recent-onset schizophrenia (41). This finding probably indicated an improved cortical function after antipsychotic treatment and future studies are warranted to confirm our findings.

### Limitations

There are several limitations in the present study. First, our study used small samples, which have been shown to yield unstable results (42, 43). Second, selection bias must be considered; this study used data from a tertiary hospital, and therefore the results may not be generalized. Third, the effects of medication on brain function should be considered. Although FES patients in the present study were minimally treated with antipsychotic medication, previous study demonstrated that even short-term treatment with antipsychotics was associated with structural brain changes (44). Fourth, there were only training and validation groups in our analysis, failure to test performance on additional independent samples (i.e., testing group) may limit the interpretation of our results. Future studies recruiting larger numbers of subjects from multi-sites are warranted. Fifth, NIRS data used in training the DL algorithm applied binary labels (FES or HCs). This dichotomous classification is widely used in researches of ML or DL, but it can be a barrier to applying this methodology in clinical practice. Most psychiatric diseases have a continuous spectrum and psychiatric comorbidities are common in a patient. The effects of psychiatric comorbidities on the brain function were not considered in the present study. In conclusion, in the present study, we distinguished FES from HCs by applying DNN to analyze frontotemporal activities during VFT measured by fNIRS and demonstrated fair sensitivity and specificity. However, additional independent datasets are needed to confirm the validity of our model.

## Data Availability Statement

The raw data supporting the conclusions of this article will be made available by the authors, without undue reservation.

## Ethics Statement

The studies involving human participants were reviewed and approved by Institutional Review Board, Taichung Veterans General Hospital. The patients/participants provided their written informed consent to participate in this study.

## Author Contributions

P-HC designed the study, managed the literature searches, performed the NIRS measurements, assessments of study subjects, statistical analyses and wrote the first draft of the manuscript. Y-HY, R-XZ, and Y-LL revised the section Deep neural network, Support vector machines, and Statistical analysis of the manuscript, and performed SVM and DNN analyses supervised by T-TL. All authors interpreted the results, revised the manuscript and approved the final version submitted for publication.

## Conflict of Interest

Y-HY, R-XZ, and Y-LL are currently employed by company MediaTek Inc. The remaining authors declare that the research was conducted in the absence of any commercial or financial relationships that could be construed as a potential conflict of interest.
